# Assigning Israeli medical graduates to internships

**DOI:** 10.1186/2045-4015-4-6

**Published:** 2015-03-20

**Authors:** Slava Bronfman, Avinatan Hassidim, Arnon Afek, Assaf Romm, Rony Shreberk, Ayal Hassidim, Anda Massler

**Affiliations:** Department of Computer Science, Bar-Ilan University, Ramat-Gan, Israel; Ministry of Health and Sackler Faculty of Medicine, Tel-Aviv University, Tel-Aviv, Israel; Harvard Business School, Cambridge, MA United States; Hadassah - Hebrew University Medical Center, Jerusalem, Israel; Shaare Zedek Medical Center, Jerusalem, Israel; Schneider Children’s Medical Center, Petach-Tikvah, Israel

**Keywords:** Internship, Lottery, Market design, Israel, Medical graduates

## Abstract

**Background:**

Physicians in Israel are required to do an internship in an accredited hospital upon completion of the medical studies, and prior to receiving the medical license. For most students, the assignment is determined by a lottery, which takes into consideration the preferences of these students.

**Objectives:**

We propose a novel way to perform this lottery, in which (on average) a larger number of students gets one of their top choices. We report about implementing this method in the 2014 Internship Lottery in Israel.

**Methods:**

The new method is based on calculating a tentative lottery, in which each student has some probability of getting to each hospital. Then a computer program “trades” between the students, where trade is performed only if it is beneficial to both sides. This trade creates surplus, which translates to more students getting one of their top choices.

**Results:**

The average student improved his place by 0.91 seats.

**Conclusions:**

The new method can improve the welfare of medical graduates, by giving them more probability to get to one of their top choices. It can be applied in internship markets in other countries as well.

## Background

Variances in teaching and professional guidance, the prospect of performing the residency in the same hospital, social interests and different work terms all contribute to the importance of where a medical graduate will perform his year of internship. In Israel, despite its relatively small size, location also plays a major role in students’ preferences, and their choice among the 23 geographically-scattered hospitals which receive interns is often influenced by this criterion. The importance of this decision led to the establishment of student-run internship committee to which representatives are elected annually. The committee is responsible for setting the rules of the internship lottery and prioritizing certain populations such as outstanding students, PhD students, couples and parents.

In the past couple of decades, this committee converged to a method known as *Random Serial Dictatorship* (henceforth RSD)^a^. This mechanism randomly chooses an ordering over students, and then the students take turns in picking the hospital they want most among those which still have not met their capacity. In Israel, up until a few years ago when the system was computerized, students physically gathered in a large auditorium and ID numbers were drawn out of a hat. This method gives each intern some probability of being assigned to any of the hospitals, depending on his place in the ordering, on the decision of the interns who were drawn before him, and his own preferences.

While any allocation selected by RSD is ex-post Pareto-efficient^b^, there are trade opportunities prior to conducting the lottery. This paper describes the trade opportunities, and how to utilize them to everyone’s benefit.

We have applied this mechanism to assign medical students to internships in Israel of the class of 2014. This required approval by the ministry of health, by the students’ internship committee, and by a ballot cast by the students, in which the mechanism described hereafter would achieve an absolute majority of the votes (the mechanism ended gaining the support of 80% of the voters and 55% of the students). This paper presents the results of the implementation.

## Methods

We know that after the assignment is done, there is no room for trade. Still, we want to let the students trade to achieve a better allocation. Therefore, instead of trading seats in hospitals, we trade probability shares. Indeed, a student *i* attending the lottery should not care too much about the internal mechanics − all he should care about is *p*_*i*,1_,…*p*_*i*,23_ where *p*_*i*,*k*_ is the probability student *i* gets his *k*’th choice.

Looking at the probability vector each student gets, RSD turns out to be far from optimal. Consider for example the following fictitious lottery, which involves four students and four hospitals, each with capacity 1 [Table 1].

**Table 1:** **The four students’ prioritized preferences over hospitals**

Alice	Diane	Bob	Charlie
A	A	A	A
B	B	B	B
C	C	D	D
D	D	C	C

All students ranked hospitals A and B as their first or their second choice respectively. Alice and Diane each ranked hospital C as their third choice and then hospital D, while Bob and Charlie each ranked hospital D as their third choice and C as their fourth.

We analyze the probability that Alice gets each hospital in this example:

- With probability  she is the first to choose. In this case she chooses *A*, and therefore .
- With probability  she is the second to choose. In this case *A* is already taken, and therefore she chooses *B*. Therefore .
- With probability  she is the third. In this case, *A* and *B* are already taken, and Alice chooses *C*. But this is not the only way Alice can get *C*. If Alice is the last to choose, then it is possible that *C* is still open, and she can take it. Indeed, if Diane goes first and takes *A*, Bob goes second and takes *B*, Charlie is third and takes *D*, then Alice can get *C* although she is the last to choose. Looking at this more closely, one can see that if Alice is fourth and Diane is not the third, Alice would also get *C*. This means that the total probability that Alice gets *C* is .
- With probability , Alice is fourth, and Diane was third. This is the only case in which Alice gets *D*, and therefore .

One can verify that the sum of probabilities is 1, and Alice is always assigned to a hospital.

A similar argument shows that Bob has probability of  to get *A*, probability of  to get *B*, probability of  to get *D* and probability of  to get *C*.

Note that in this simple example, Alice and Bob could trade probabilities, and this would benefit both of them. Imagine that Bob could somehow give Alice his probability of being assigned to *C*, and in return she would give him her probability of being assigned to *D*. This would result in a state in which Alice has probability of  for *A*,  for *B* and  for *C*. Bob would have probability of  for *A*,  for *B* and  for *D*, which is an improvement for both of them, compared to the RSD probability shares. Charlie and Diane could trade probabilities among themselves in a similar manner.

**Figure 1:**
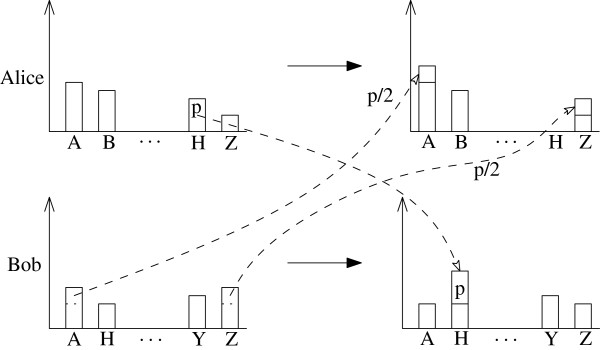
**Alice gives Bob her probability of being assigned to** ***H*** **, and in return she gets half of this probability to her first choice and half of it to her last choice from Bob.**

While this already improves the current state of affairs, we can do even better. Suppose that Alice and Bob agree on their first and tenth choices, but Bob’s second choice is some hospital *H*, which is also Alice’s ninth choice. Also, suppose that Alice has some positive probability *p* of being assigned to *H*. In this case both students would possibly be happier if Alice “gives” Bob her probability *p* of getting to *H*, and Bob would “give” her  probability of getting to his first place, and  probability of getting to his tenth place (see Figure 1). In this case:

1. Assuming there is no huge gap between the ninth place and the tenth place, Alice should be happy. She “lost” probability *p* of getting to her ninth place, and received probability of  to get to her tenth place (similar), and probability of  to get to her first place.
2. Assuming there is no huge gap between the first and second place, Bob should be happy. He lost  probability from his first place and  probability from his tenth place, to get *p* probability for his second place.

While clearly this trade is beneficial, it raises a couple of subtle points, which are related:

1. Why should Bob give  of his tenth place and  of his first place? Why not  of his tenth place, and  of his first place, or vice verse, or some different numbers?
2. The difference between first and second place is usually larger than that between ninth and tenth place.

As explained below, the students were asked to fill surveys, to assert the difference between the first and the second place, the second and the third place and so on. Based on the surveys results’, more weight was given to the difference between first and second place than to the difference between the ninth and the tenth.

One question which was not addressed in these simple examples is how to decide which students should trade with whom, and what trades to perform. Of course, the students do not actually trade with each other, but rather a computer program “virtually” trades on their behalf. We also use more complex trades, which may involve three or more students at once, if they benefit all the participants.

Another question is how do we perform the lottery at the end of the process? With RSD, we could just choose an ordering over the students. But in the first example, what lottery gives Alice probability of  to get to *C* and Bob probability  to get to *D*? Note that if each student would just select a random hospital according to the probabilities, it is possible that two students would be assigned to the same hospital, so this is not a valid solution.

In the rest of the section, we formally explain our method, and solve the two questions presented above.

### Description of the new lottery

Our method works as follows. First, approximate the probability of each student to be assigned to each hospital using RSD. We do this by running a large number of trials, *N*, and by the law of large numbers the average of all those RSD lotteries will be sufficiently close to the true value. The probability is calculated as
1

where *n*_*i*,*k*_ is the number of RSD lotteries in which Student *i* was assigned to the hospital he ranked as his *k*-th choice.

Once we have the approximated probabilities we continue with the second stage of the algorithm which is trading the probabilities among the students. We do this using *Linear Programming*, which is a mathematical optimization method for maximizing a target function subject to several constraints [1]^c^. In our interns assignment problem there are two constraints:

1. Each hospital has an upper limit for the number of interns that can be assigned to it. This *capacity constraint* is determined by the Ministry of Health.
2. No student is worse off compared to what he would have got under RSD. This *individual rationality constraint* is enforced by defining the *happiness* of each student from his vector of probabilities, and then requiring that for every student individually the happiness can not decrease by the trading stage (intuitively if this would decrease his happiness the student would not trade).

As for the target function, we want to maximize the total satisfaction of the students after trading. The full description of the constraints and the target function appears in Appendix.

After the optimal probabilities have been acquired, we only need to randomize an allocation according to these probabilities. This, however, is not an easy process, as we want the lottery over valid allocations to respect all the interns’ probability allocations simultaneously. Fortunately, the Birkhoff-von Neumann theorem provides a solution to this problem [2, 3]. In order to apply the theorem for the allocation problem, we represent the probabilities data we have until this stage with a matrix of size *n*×*m*, where the rows are the interns, the columns are the hospitals, and cell (*i*,*j*) represents Student *i*’s probability to be assigned to Hospital *j*. The theorem ensures that any random assignment of the objects in the rows of the matrix to the objects in the columns can be implemented. Furthermore, Birkhoff’s proof provides a constructive algorithm for the implementation [4]. Using an extension of this theorem we can create a lottery which respects the improved probabilities gained by trading [5].

## Results and discussion

**Figure 2:**
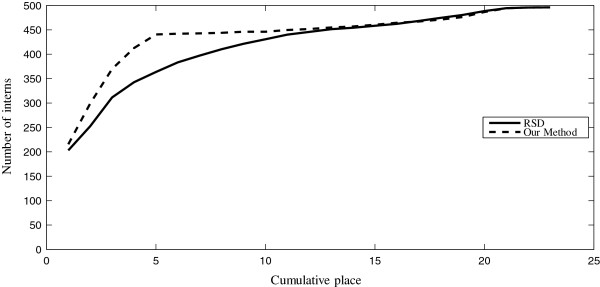
**The number of interns as a function of being assigned one of their top choices.** The solid line shows the results of RSD, and the dashed line shows the result of the new method.

Figure 2 depicts the number of students who were assigned to one of their top *k* choices as a function of *k*. The gap between the two curves shown in the figure (the area under the dashed curve) represents the improvement of the new method compared to RSD. While RSD would assign 203 interns to their first choice hospital, 50 to the second and 59 to the third, the new method assigns 216 interns to their first choice, 84 to their second choice and 70 to the third choice.

Furthermore, using our data we would like to rate hospitals, according to how high the interns ranked them. Such a rating can be useful, since it gives the hospitals a better picture of their status, and raises a red flag if a specific hospital should improve the way it treats its interns or signals quality to the Ministry of Health. Before we aggregate the data to create such a rating, it is useful to look at the ranking distribution of a specific hospital, and see if it makes sense.

**Figure 3:**
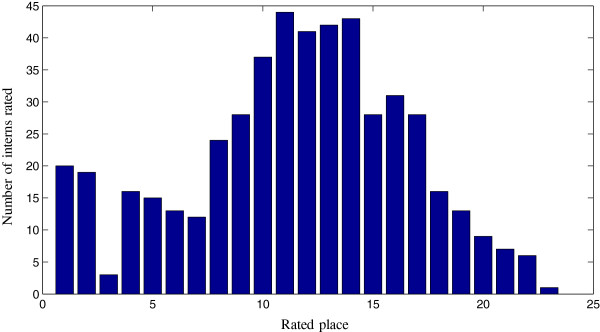
**The number of interns who ranked** ***Hadassah*** **hospital in each place.**

For example, Hadassah Medical Center’s ranking distribution is depicted in Figure 3. About 10*%* of the interns ranked Hadassah as one of their top three choices, which possibly indicates that they are residents of Jerusalem and that location is important to them. The rest of the students ranked Hadassah around the middle of their rank order list, suggesting that interns consider Hadassah to be a good hospital, and that the demand for being an intern there is quite high.

**Figure 4:**
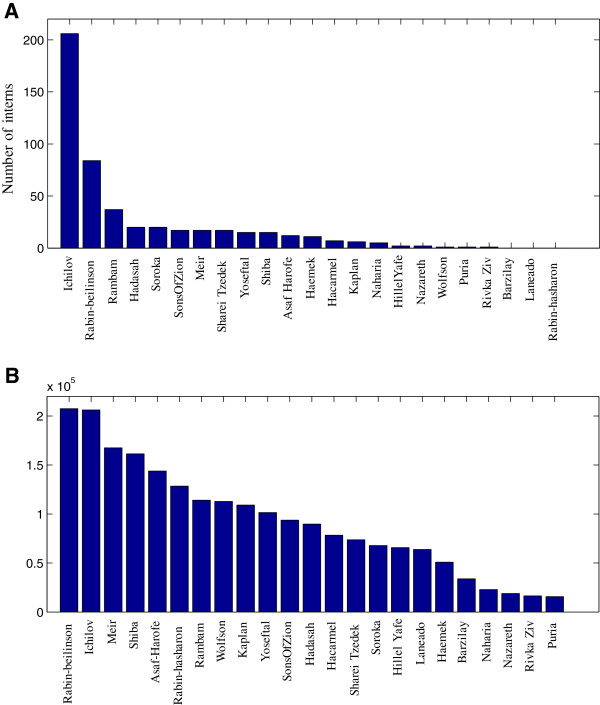
**A comparison between two rating approaches.** **(A)** The number of interns who ranked the given hospital as their first choice. **(B)** The calculated value of the given hospital according to the survey-based weights.

We now aggregate the rankings of all students, to create a rating across hospitals. We compare two methods of rating the hospitals. The first method is to use the same weights that we used when defining students’ happiness, as described in Eq. 2^d^. The second method is the traditional rating of hospitals in this lottery, which is based on the number of interns who ranked a specific hospital as their first choice. Figure 4 demonstrates that our new rating approach provides very different results from the traditional rating approach, and it is perhaps more advisable to use it as it takes into account the entire rank order lists of all students.

Comparing the two ratings, one can see several differences:

1. In the traditional approach, Rabin-Hasharon comes out last, although Rabin-Beilinson came in second. The reason that this happens, is that every student rates Rabin-Belinson above Rabin-Hasharon, so Rabin Hasharon is never first. However, the difference is very small - they are both campuses of the Rabin Hospital, and are 10 minutes apart^e^. In the new rating, Belinson comes in first (but almost at a tie with Ichilov), and Hasharon comes sixth.
2. The last hospitals in the traditional rating have very low scores, and a single student who changes his vote can change the rating. The new rating is much more robust.
3. The decay in the score makes more sense in the second rating. It is never too sharp, and there is no huge difference between the first two places.

We take the new rating as further evidence that the values at which the algorithm trades probabilities make sense.

## Conclusion

In this paper we presented a novel technique to perform assignments, and showed that it greatly improved the assignment of Israeli medical graduates to internships, increasing the number of students who received one of their top choices. This method requires the medical students to “trade” probabilities to get to different places, and therefore creates a new comparison between different hospitals, based on how much they are desired in the trade. We presented the results of this comparison, and showed that it makes much more sense than the traditional one (namely order the hospitals according to the number of students who ranked them first). Seeing that the new rating makes sense is an evidence that the probabilities in the new lottery are traded correctly.

A similar lottery can be applied to other countries with an internship matching process in which future interns belonging to the same category or rank have identical priority (e.g., Australia and Ireland). Other countries that strictly rank all the interns (such as France) cannot benefit from the suggested algorithm. Similarly, in countries in which hospitals also have preferences over doctors, a better treatment will be using a suitable two-sided matching algorithm (e.g., the residency matches in UK and USA). Our approach may be extended to other realms, such as allocating seats in university courses. However, each application could require some modifications to the algorithm, depending on the specifics of the domain.

## Endnotes

^a^ Often referred to also as “Random Priority”. See, for example, [6].

^b^ Meaning that no trade can happen right after the allocation. Indeed if Alice and Bob would like to swap places after RSD is performed, and Alice chose her hospital before Bob, then why didn’t she choose Bob’s hospital? A mathematically equivalent way of saying this is that no student can get a better hospital without harming a different student. Note that it is possible that preferences change between the time of the lottery, and the actual start of residency, and then trade may be beneficial for both sides − however, right after the lottery is over students should not want to trade.

^c^ Similar approaches to object assignment have been already suggested. See, for example, [7] and [8].

^d^ The rating we get using this method is very similar to the rating one gets using the more familiar Borda Count method.

^e^ In previous years they appeared together in the internship forms under “Rabin” and a second lottery was performed between the students to see which student goes to which campus.

## Appendix

### Linear programming technical explanation

To represent the *individual rationality constraint*, Let *m* be the number of hospitals, we define Student *i*’s happiness prior to the trading stage as
2

i.e., a function that represents students’ strong preference to get to their top ranked hospitals. If the probabilities we get after the trading stage are  (as defined in Eq. 1), then we define the happiness following the optimization as
3

Now our constraints are  for every student *i*.

Regarding the target function, as described in the Methods section, we want to maximize the total satisfaction of the students after trading. Letting *n* denote the number of students (in 2014, *n*=496), our optimization goal is going to be
4

that is, maximizing the sum of happiness for all interns. This target function, as well as the definition of happiness in Eq. 2, was chosen following a survey filled by approximately 70 interns and 6th year medical students. We note that using similar target functions and making small changes in the weights used to define happiness did not have a profound effect on the statistics of the assignment.

## Applying the algorithm in the Israeli internship market

One of the challenges we faced was how to introduce the algorithm to the Israeli market. While there is a standard procedure for medical clinical trials, here there are no patients involved, so it is less clear what experiments should be made. To use the new algorithm, we needed the agreement of two entities: the Ministry of Health and the student body.

Convincing the Ministry of Health to participate was relatively easy, since the Ministry of Health is happy to try out new things. If an idea is not working well, one class would suffer, but if an idea improves the system the gain can affect future classes of students as well. While the Ministry of Health is formally assigning students to internships, for over a decade it has delegated the decision of which student goes where to the students themselves.

Convincing the medical graduates to use the new mechanism was more complicated. Each medical graduate has a "single experiment" (his own outcome), and sees no personal gain in improving the system for future generations. Moreover, the happiness function assumes a certain distance between the first place to the second, the second to the third and so on. If a student strongly disagrees with the happiness function (e.g., the student is absolutely indifferent between his top eight places but hates all the rest) the new mechanism may not serve him well. The student internship committee decided that there will be a poll on whether the mechanism should be changed, and the new mechanism needs to get an absolute majority (a majority of the students should vote for the change and not just a majority of the voters). The poll was conducted, and 80% of the voters (55% of the students) preferred the LP based mechanism. Talking to students and explaining them the mechanism, we were impressed to see how well they understood the mathematical notions behind it, and their openness to new ideas.

## Authors’ information

Anda Massler received her MSc. in microbiology and her M.D. in 2009, both from the Hebrew University. She is now a resident in child psychiatry at the Shneider Medical Center. Ayal Hassidim received his M.D. from Ben Gurion University in 2009. After serving in the IDF medical corps, he started his residency in general surgery at the Shaare Zedek Medical Center. Rony Shreberk received her M.D. from Tel Aviv University in 2013. She is now a resident in the Department of Dermatology, Hadassah - Hebrew University Medical Center. Arnon Afek is a professor at the Sackler Medical School at Tel Aviv University, and is the director-general of the Ministry of Health. Slava Bronfman graduated from the Technion in 2010. He is now a student of computer science at Bar-Ilan University. Avinatan Hassidim is an assistant professor of computer science, in Bar Ilan University. His research focuses on Algorithmic Game Theory and Market Design. Assaf Romm is a PhD student of Business Economics at Harvard University, and his research focus is market design and matching systems. He holds a B.Sc. in Mathematics, Economics and Humanities and an MA in Economics and the Study of Rationality from the Hebrew University of Jerusalem, and an MA in Business Economics from Harvard University.
